# Complete genome sequence of *Aminobacterium colombiense* type strain (ALA-1^T^)

**DOI:** 10.4056/sigs.902116

**Published:** 2010-06-15

**Authors:** Olga Chertkov, Johannes Sikorski, Evelyne Brambilla, Alla Lapidus, Alex Copeland, Tijana Glavina Del Rio, Matt Nolan, Susan Lucas, Hope Tice, Jan-Fang Cheng, Cliff Han, John C. Detter, David Bruce, Roxanne Tapia, Lynne Goodwin, Sam Pitluck, Konstantinos Liolios, Natalia Ivanova, Konstantinos Mavromatis, Galina Ovchinnikova, Amrita Pati, Amy Chen, Krishna Palaniappan, Miriam Land, Loren Hauser, Yun-Juan Chang, Cynthia D. Jeffries, Stefan Spring, Manfred Rohde, Markus Göker, James Bristow, Jonathan A. Eisen, Victor Markowitz, Philip Hugenholtz, Nikos C. Kyrpides, Hans-Peter Klenk

**Affiliations:** 1 DOE Joint Genome Institute, Walnut Creek, California, USA; 2 Oak Ridge National Laboratory, Oak Ridge, Tennessee, USA; 3 DSMZ – German Collection of Microorganisms and Cell Cultures GmbH, Braunschweig, Germany; 4 Los Alamos National Laboratory, Bioscience Division, Los Alamos, New Mexico, USA; 5 Biological Data Management and Technology Center, Lawrence Berkeley National Laboratory, Berkeley, California, USA; 6 HZI – Helmholtz Centre for Infection Research, Braunschweig, Germany; 7 University of California Davis Genome Center, Davis, California, USA

**Keywords:** strictly anaerobic, fermentation of amino acids, gram-negative firmicute, syntrophic organism, *Synergistaceae*, GEBA

## Abstract

*Aminobacterium colombiense* Baena *et al.* 1999 is the type species of the genus *Aminobacterium*. This genus is of large interest because of its isolated phylogenetic location in the family *Synergistaceae*, its strictly anaerobic lifestyle, and its ability to grow by fermentation of a limited range of amino acids but not carbohydrates. Here we describe the features of this organism, together with the complete genome sequence and annotation. This is the second completed genome sequence of a member of the family *Synergistaceae* and the first genome sequence of a member of the genus *Aminobacterium*. The 1,980,592 bp long genome with its 1,914 protein-coding and 56 RNA genes is part of the *** G****enomic* *** E****ncyclopedia of* *** B****acteria and* *** A****rchaea * project.

## Introduction

Strain ALA-1^T^ (= DSM 12261) is the type strain of the species *Aminobacterium colombiense*, which is the type species of the genus *Aminobacterium* [1,2]. The name of the genus relates to its ability to ferment amino acids and the species name refers to origin of the isolate, Columbia [1]. Currently, the genus *Aminobacterium* consists of only two species [1,3,4]. Strain ALA-1^T^ has been isolated from an anaerobic dairy wastewater lagoon in 1998 or before [1]. At the moment, strain ALA-1^T^ is the only known isolate of this species. Highly similar (98%) nearly complete (>1,400 bp) uncultured 16S gene clone sequences were frequently obtained from anaerobic habitats, *e.g*., from anaerobic municipal solid waste samples in France [5], from a biogas fermentation enrichment culture in China (GU476615), from a swine wastewater anaerobic digestion in a UASB reactor in China (FJ535518), and from a mesophilic anaerobic BSA digester in Japan [6], suggesting quite a substantial contribution of *Aminobacterium* to anaerobic prokaryotic communities. The type strain of the only other species in the genus*,* *A. mobile* [3] shares 95% 16S rRNA sequence identity with *A. colombiense*, whereas the type strains of the other species in the family *Synergistaceae* share between 84.3 and 88.3% 16S rRNA sequence identity [7]. Environmental samples and metagenomic surveys detected only one significantly similar phylotype (BABF01000111, 92% sequence similarity) in a human gut microbiome [7], with all other phylotypes sharing less than 84% 16S rRNA gene sequence identity, indicating a rather limited general ecological importance of the members of the genus *Aminobacterium* (status April 2010). Here we present a summary classification and a set of features for *A. colombiense* ALA-1^T^, together with the description of the complete genomic sequencing and annotation.

## Classification and features

**Figure 1:**
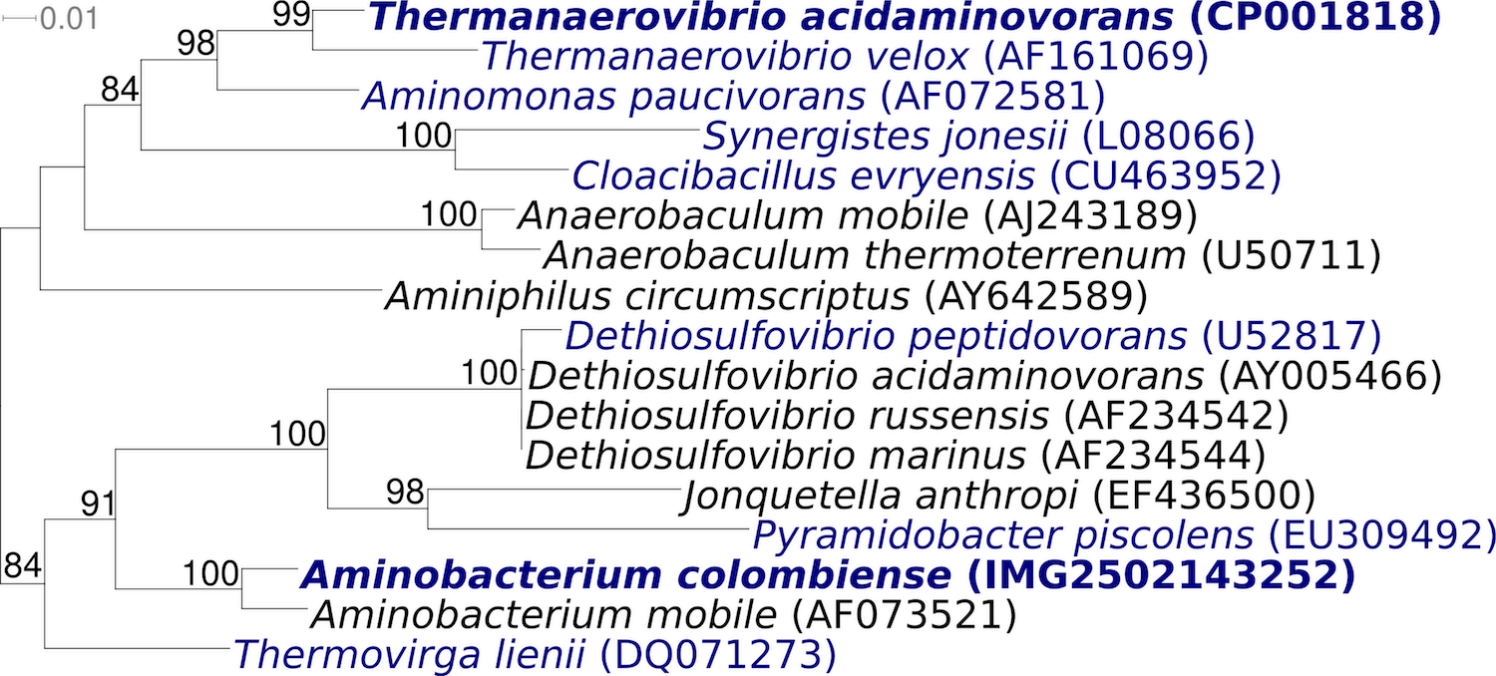
Phylogenetic tree highlighting the position of *A. colombiense* ALA-1^T^ relative to the other type strains within the phylum *Synergistetes*. The tree was inferred from 1,282 aligned characters [8,9] of the 16S rRNA gene sequence under the maximum likelihood criterion [10] and rooted in accordance with the current taxonomy [11]. The branches are scaled in terms of the expected number of substitutions per site. Numbers above branches are support values from 250 bootstrap replicates [12] if larger than 60%. Lineages with type strain genome sequencing projects registered in GOLD [13] are shown in blue, published genomes in bold, *e.g*. the recently published GEBA genome of *Thermanaerovibrio acidaminovorans* [14].

Figure 1 shows the phylogenetic neighborhood of *A. colombiense* ALA-1^T^ in a 16S rRNA based tree. The sequences of the three identical copies of the 16S rRNA gene in the genome differ by 14 nucleotides (0.9%) from the previously published 16S rRNA sequence generated from DSM 12661 (AF069287). which contains 3 ambiguous base calls. These differences are most likely due to sequencing errors in AF069287.

**Figure 2:**
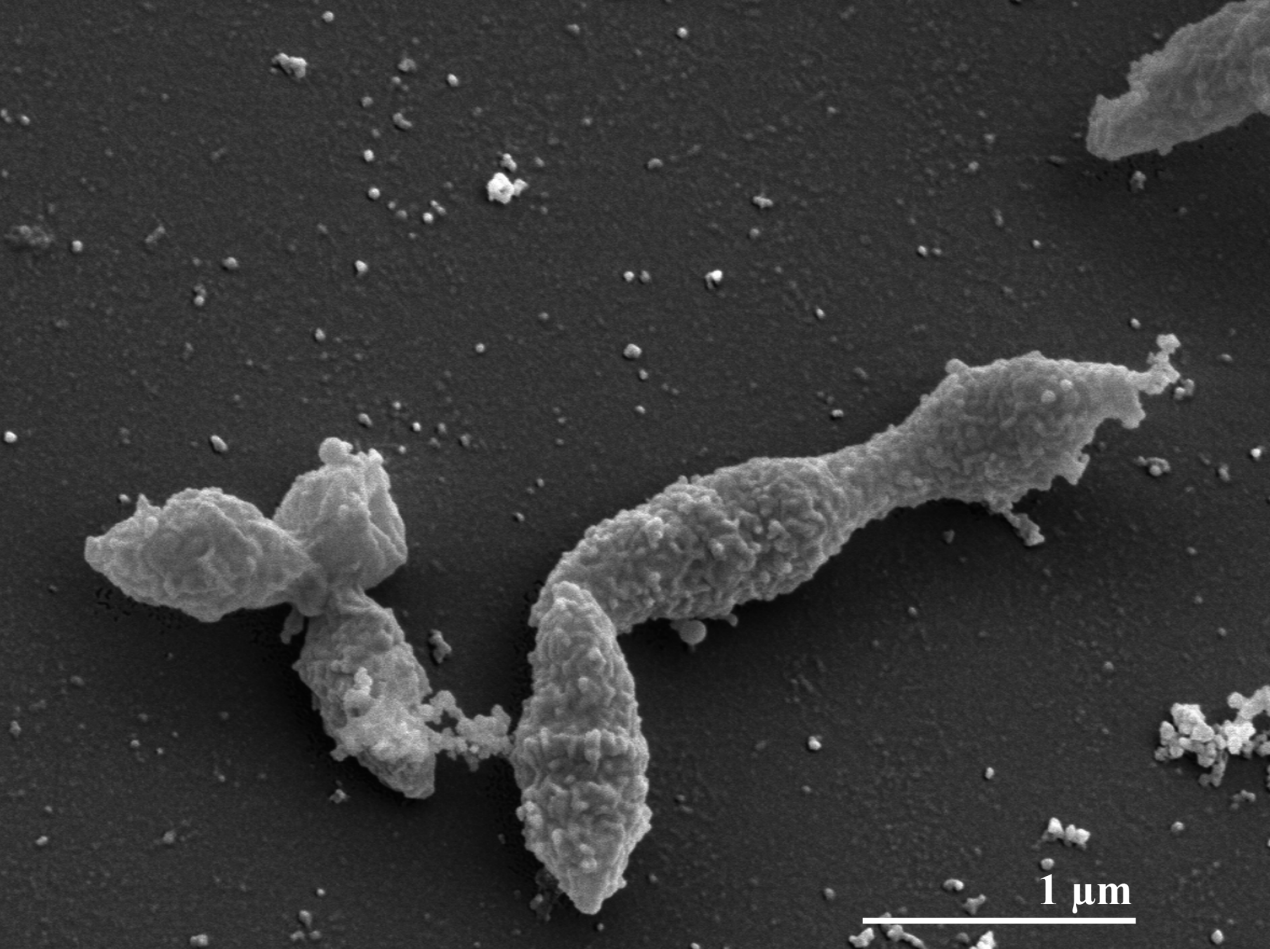
Scanning electron micrograph of *A. colombiense* ALA-1^T^

The cells are rod-like, occasionally slightly curved with 3-4 µm in length and 0.2-0.3 µm in width (Figure 2 and Table 1) [1]. The colonies are up to 1.0 mm in diameter and are round, smooth, lens-shaped, and white [1]. Strain ALA-1^T^ requires yeast extract for growth and ferments serine, glycine, threonine, and pyruvate in its presence [1]. Poor growth is obtained on casamino acids, peptone, biotrypcase, cysteine and α-ketoglutarate [1]. The fermentation and end-products include acetate and H_2_, and also propionate in the case of α-ketoglutarate fermentation. Carbohydrates (such as glucose, saccharose, ribose, xylose, cellobiose, mellobiose, maltose, galactose, mannose, arabinose, rhamnose, lactose, sorbose and mannitol), gelatin, casein, glycerol, ethanol, acetate, propionate, butyrate, lactate, citrate, fumarate, malate, succinate and the other amino acids tested are not utilized [1].

**Table 1:** Classification and general features of *A. colombiense* ALA-1^T^ according to the MIGS recommendations [15]

**MIGS ID**	**Property**	**Term**	**Evidence code**
	Current classification	Domain *Bacteria*	TAS [16]
		Phylum *Synergistetes*	TAS [17]
		Class *Synergistia*	TAS [17]
		Order *Synergistales*	TAS [17]
		Family *Synergistaceae*	TAS [17]
		Genus *Aminobacterium*	TAS [1,18]
		Species *Aminobacterium colombiense*	TAS [1,18]
		Type strain ALA-1	TAS [1]
	Gram stain	negative	TAS [1]
	Cell shape	slightly curved to rod shaped	TAS [1]
	Motility	nonmotile	TAS [1]
	Sporulation	non-sporulating	TAS [1]
	Temperature range	mesophile, 20°C – 42°C, no growth at 18°C and 45°C	TAS [1]
	Optimum temperature	37 °C	TAS [1]
	Salinity	no NaCl required, tolerates less than 1.5% NaCl	TAS [1]
MIGS-22	Oxygen requirement	strictly anaerobic	TAS [1]
	Carbon source	serine, threonine, glycine and pyruvate, not carbohydrates	TAS [1]
	Energy source	serine, threonine, glycine and pyruvate, not carbohydrates	TAS [1]
MIGS-6	Habitat	anaerobic sludge	TAS [1]
MIGS-15	Biotic relationship	free-living	TAS [1]
MIGS-14	Pathogenicity	pathogenicity is not reported	NAS
	Biosafety level	1	TAS [19]
	Isolation	anaerobic dairy wastewater lagoon	TAS [1]
MIGS-4	Geographic location	Santa Fe de Bogota, Colombia	TAS [1]
MIGS-5	Sample collection time	1998 or before	TAS [1]
MIGS-4.1 MIGS-4.2	Latitude Longitude	4.63 -74.08	NAS
MIGS-4.3	Depth	unknown	
MIGS-4.4	Altitude	about 2,640 m	NAS

Evidence codes - IDA: Inferred from Direct Assay (first time in publication); TAS: Traceable Author Statement (i.e., a direct report exists in the literature); NAS: Non-traceable Author Statement (i.e., not directly observed for the living, isolated sample, but based on a generally accepted property for the species, or anecdotal evidence). These evidence codes are from of the Gene Ontology project [20]. If the evidence code is IDA, then the property was directly observed for a live isolate by one of the authors or an expert mentioned in the acknowledgements.

As typical for anoxic habitats, strain ALA-1^T^ is engaged in syntrophic interactions: alanine, glutamate, valine, isoleucine, leucine, methionine, aspartate and malate are oxidized only in the presence of the hydrogenotroph, *Methanobacterium formicicum,* strain DSM 1525 [1]. In addition, the utilization of cysteine, threonine and α-ketoglutarate are also improved in the presence of *M. formicicum* [1]. An 80% hydrogen atmosphere (supplied as H_2_-CO, (80:20) at 2 bar pressure) inhibits growth of strain ALA-1^T^ on threonine and α-ketoglutarate, whereas glycine degradation is not affected [1]. Serine and pyruvate degradation are partially affected by the presence of hydrogen. Sulfate, thiosulfate, elemental sulfur, sulfite, nitrate, and fumarate are not utilized as electron acceptors [1]. Strain ALA-1^T^ does not perform the Stickland reaction when alanine is provided as an electron donor and glycine, serine, arginine or proline are provided as electron acceptor.

As noted above, alanine is oxidized only in the presence of the hydrogenotroph *M. formicicum*, which utilizes the produced H_2_ [1]. In the absence of an H_2_-consuming organism, the H_2_ partial pressure would rapidly reach a level that thermodynamically inhibits further fermentation [21]. Adams and colleagues used a H_2_-purging culture vessel to replace the H_2_-consuming syntrophic partner, in order to study in detail the energetic characteristics of alanine consumption of strain ALA-1^T^ in a pure culture [21].

Strain ALA-1^T^ is non-motile [1], whereas interestingly the other species in the genus, *A. mobile*, is motile by means of lateral flagella [3]. A parallel situation is in the genus *Anaerobaculum* (Figure 1), where *A. thermoterrenum* is non-motile [22] but *A. mobile* is motile by means of lateral flagella [23]. In fact, the phenotype of non-motility versus motility by means of lateral flagella is heterogeneously distributed among the organisms depicted in Figure 1. This may suggest that the last common ancestor of the group shown in Figure 1 was motile by flagella and that the selection pressure for a functioning flagella might be currently more relaxed in this group, leading in individual strains to mutational inactivation of the flagella. Interestingly, the annotation of the genome does not give any indication of the presence of any genes related to flagellar assembly. The only genes related to cellular motility refer to type II secretory pathway and to pilus assembly. This is surprising, as it is hardly probable that strain ALA-1^T^ lost all genes for flagellar assembly after the evolutionary separation of strain ALA-1^T^ and its closely related sister species *A. mobile* from their last common ancestor. A similar situation has been observed in the non-motile strain *Alicyclobacillus acidocaldarius* 104-IA^T^ in comparison to several motile sister species in the genus *Alicyclobacillus* [24]. Here, the genome of the non-motile strain *A. acidocaldarius* 104-IA^T^ still contains most of the genes needed for flagellar assembly [24]. Thus, the genotypic status of flagellar motility in the genus *Aminobacterium* remains unclear.

### Chemotaxonomy

Ultrathin sections of strain ALA-1^T^ revealed a thick cell wall with an external S-layer similar to that of Gram-positive type cell walls [1]. Unfortunately, no chemotaxonomic data have been published for the genus *Aminobacterium*. Among the organisms depicted in Figure 1, chemotaxonomic data are available for *Dethiosulfovibrio peptidovorans*, *Jonquetella anthropi*, *Pyramidobacter piscolens*, *Cloacibacillus evryensis*, and *Synergistes jonesii*, though the data are not always present in the original species description publications [25-27]. In major phenotypes, such as being strictly anaerobic and Gram-negative in staining within the usually Gram-positive *Firmicutes*, mostly also in their ability to degrade amino acids, the organisms shown in Figure 1 are highly similar, which may justify also a comparison in their chemotaxonomic features. The major fatty acids in different strains of *Jonquetella* are iso-C_15:0_ (25-43%) and C_16:0_ (14-21%), other iso-branched and unbranched fatty acids are present in smaller amounts, and anteiso-C_15:0_ is below 5% [26]. In *Dethiosulfovibrio*, the major fatty acid is iso-C_15:0_ (59.7%), followed by C_18:0_ (9.0%) and C_16:0_ (8.5%) [26]. *Dethiosulfovibrio* differs qualitatively from *Jonquetella* by the absence of anteiso branched fatty acids and by the presence of C_18:1_ω9c (3.0%) [26]. The major fatty acids in two strains of *P. piscolens* are C_14:0_ (16-19%) and C_13:0_ (12-14%) [27]. The cellular fatty acids of *C. evryensis* are characterized by a mixture of saturated, unsaturated, hydroxy- and cyclopropane fatty acids [25]. The major fatty acids were iso-C_15:0_ (16.6%), iso-C_15:0_ 3-OH (12.4%) and C_17:1_ω6c (9.5%) [25]; the major fatty acids in its closest relative, *Synergistes jonesii*, were C_15:0_ (16.0%), C_20_cyc (14:0) and C_17:1_ω6c (9.0%) [25]. The polar fatty acid profile of *C. evryensis* (data not shown in the original publication) revealed diphosphatidylglycerol, phosphatidylglycerol, phosphatidyl-ethanolamine and phosphatidylmonomethylamine [25].

## Genome sequencing and annotation

### Genome project history

This organism was selected for sequencing on the basis of its phylogenetic position [28], and is part of the *** G****enomic* *** E****ncyclopedia of* *** B****acteria and* *** A****rchaea * project [29]. The genome project is deposited in the Genome OnLine Database [13] and the complete genome sequence is deposited in GenBank. Sequencing, finishing and annotation were performed by the DOE Joint Genome Institute (JGI). A summary of the project information is shown in Table 2.

**Table 2:** Genome sequencing project information

**MIGS ID**	**Property**	**Term**
MIGS-31	Finishing quality	Finished
MIGS-28	Libraries used	Three genomic libraries: one 454 pyrosequence standard library; one 454 12kb pyrosequence library; one Illumina 250bp library
MIGS-29	Sequencing platforms	454 GS FLX Titanium; Illumina GAii
MIGS-31.2	Sequencing coverage	85.5× 454 pyrosequence; 909 Mb Illumina data
MIGS-30	Assemblers	Newbler version 2.0.0-PostRelease-10/28/2008, phrap
MIGS-32	Gene calling method	Prodigal, GenePRIMP
	INSDC ID	CP001997
	Genbank Date of Release	April 5, 2010
	GOLD ID	Gc01257
	NCBI project ID	32587
	Database: IMG-GEBA	2502082107
MIGS-13	Source material identifier	DSM 12261
	Project relevance	Tree of Life, GEBA

### Growth conditions and DNA isolation

*A. colombiense* ALA-1^T^, DSM 12661, was grown anaerobically in DSMZ medium 846 (Anaerobic serine/arginine medium) [30] at 37°C. DNA was isolated from 1-1.5 g of cell paste using MasterPure Gram Positive DNA Purification Kit (Epicentre MGP04100) adding additional 1µl lysozyme and 5 µl mutanolysin to the standard lysis solution for 40 min incubation at 37°C.

### Genome sequencing and assembly

The genome was sequenced using a combination of Illumina and 454 technologies. An Illumina GAii shotgun library with reads of 909 Mb, a 454 Titanium draft library with average read length of 283 bases, and a paired end 454 library with average insert size of 12 kb were generated for this genome. All general aspects of library construction and sequencing can be found at http://www.jgi.doe.gov/. Draft assemblies were based on 169 Mb 454 draft data and 454 paired end data (543,550 reads). Newbler (version 2.0.0-PostRelease-10/28/2008 was used) parameters are -consed -a 50 -l 350 -g -m -ml 20. The initial Newbler assembly contained 18 contigs in 1 scaffold. The initial 454 assembly was converted into a phrap assembly by making fake reads from the consensus, collecting the read pairs in the 454 paired end library. Illumina sequencing data was assembled with VELVET [31], and the consensus sequences were shredded into 1.5 kb overlapped fake reads and assembled together with the 454 data. The Phred/Phrap/Consed software package (www.phrap.com) was used for sequence assembly and quality assessment in the following finishing process. After the shotgun stage, reads were assembled with parallel phrap (High Performance Software, LLC). Possible mis-assemblies were corrected with gapResolution (http://www.jgi.doe.gov/), Dupfinisher, or sequencing cloned bridging PCR fragments with subcloning or transposon bombing [32]. Gaps between contigs were closed by editing in Consed, by PCR and by Bubble PCR primer walks (J-F.Cheng, unpublished). A total of 113 additional Sanger reactions were necessary to close gaps and to raise the quality of the finished sequence. The error rate of the completed genome sequence is less than 1 in 100,000.

### Genome annotation

Genes were identified using Prodigal [33] as part of the Oak Ridge National Laboratory genome annotation pipeline, followed by a round of manual curation using the JGI GenePRIMP pipeline [34]. The predicted CDSs were translated and used to search the National Center for Biotechnology Information (NCBI) nonredundant database, UniProt, TIGR-Fam, Pfam, PRIAM, KEGG, COG, and InterPro databases. Additional gene prediction analysis and functional annotation was performed within the Integrated Microbial Genomes - Expert Review (IMG-ER) platform [35].

## Genome properties

**Figure 3:**
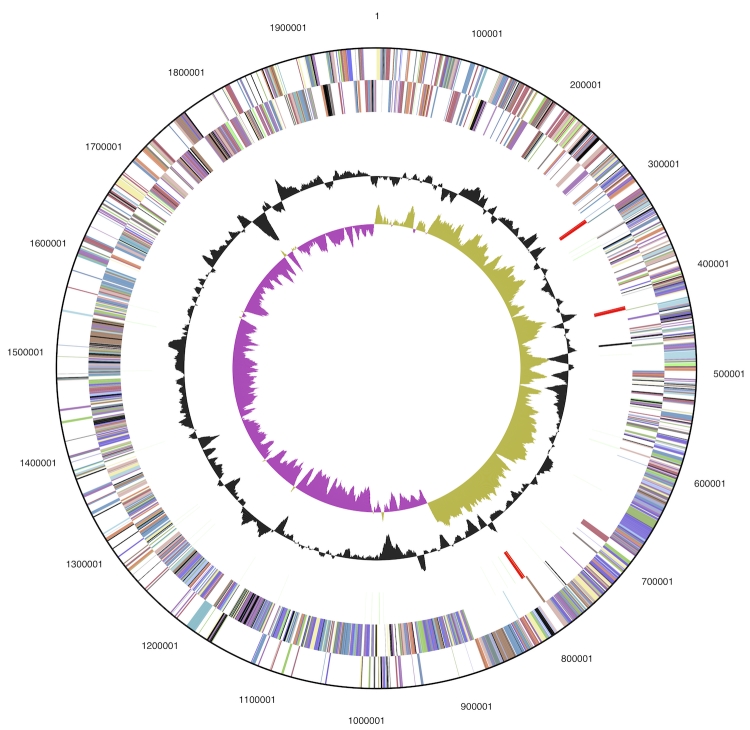
Graphical circular map of the genome. From outside to the center: Genes on forward strand (color by COG categories), Genes on reverse strand (color by COG categories), RNA genes (tRNAs green, rRNAs red, other RNAs black), GC content, GC skew.

The genome consists of a 1,980,592 bp long chromosome with an overall GC content of 45.3% (Table 3 and Figure 3). Of the 1,970 genes predicted, 1,914 were protein-coding genes, and 56 RNAs; 38 pseudogenes were also identified. The majority of the protein-coding genes (77.2%) were assigned with a putative function while the remaining ones were annotated as hypothetical proteins. The distribution of genes into COGs functional categories is presented in Table 4.

**Table 3:** Genome Statistics

**Attribute**	**Value**	**% of Total**
Genome size (bp)	1,980,592	100.00%
DNA coding region (bp)	1,837,142	92.76%
DNA G+C content (bp)	897,344	45.31%
Number of replicons	1	
Extrachromosomal elements	0	
Total genes	1,970	100.00%
RNA genes	56	2.84%
rRNA operons	3	
Protein-coding genes	1,914	97.16%
Pseudo genes	38	1.93%
Genes with function prediction	1,521	77.21%
Genes in paralog clusters	225	12.94%
Genes assigned to COGs	1,592	80.81%
Genes assigned Pfam domains	1,617	82.08%
Genes with signal peptides	337	17.11%
Genes with transmembrane helices	540	27.41%
CRISPR repeats	1	

**Table 4:** Number of genes associated with the general COG functional categories

**Code**	**value**	**%age**	**Description**
J	150	8.8	Translation, ribosomal structure and biogenesis
A	0	0.0	RNA processing and modification
K	105	6.1	Transcription
L	81	4.7	Replication, recombination and repair
B	1	0.1	Chromatin structure and dynamics
D	23	1.3	Cell cycle control, cell division, chromosome partitioning
Y	0	0.0	Nuclear structure
V	23	1.3	Defense mechanisms
T	51	3.0	Signal transduction mechanisms
M	106	6.2	Cell wall/membrane biogenesis
N	5	0.3	Cell motility
Z	0	0.0	Cytoskeleton
W	0	0.0	Extracellular structures
U	32	1.9	Intracellular trafficking, secretion, and vesicular transport
O	57	3.3	Posttranslational modification, protein turnover, chaperones
C	129	7.5	Energy production and conversion
G	118	6.9	Carbohydrate transport and metabolism
E	199	11.6	Amino acid transport and metabolism
F	66	3.9	Nucleotide transport and metabolism
H	67	3.9	Coenzyme transport and metabolism
I	42	2.5	Lipid transport and metabolism
P	98	5.7	Inorganic ion transport and metabolism
Q	26	1.5	Secondary metabolites biosynthesis, transport and catabolism
R	207	12.1	General function prediction only
S	126	7.4	Function unknown
-	378	19.2	Not in COGs
